# Registered nurses’ experiences with generative artificial intelligence: a meta-synthesis of qualitative studies

**DOI:** 10.3389/fpubh.2026.1917065

**Published:** 2026-07-27

**Authors:** Yan Deng, YiDan Zhu, JiaQi Li, Yu Xiong

**Affiliations:** 1 College of Medicine, Hunan Normal University, Changsha, China; 2 The 921st Hospital of the Joint Logistic Support Force of the Chinese People’s Liberation Army, Changsha, China

**Keywords:** digital health, generative artificial intelligence, JBI ConQual, meta-aggregation, nursing, qualitative meta-synthesis, registered nurses

## Abstract

**Objective:**

To synthesize qualitative evidence on registered nurses’ experiences of using generative artificial intelligence (GAI) in clinical practice and nursing research, and to identify perceived benefits, challenges, and support needs for its standardized implementation in nursing.

**Methods:**

A qualitative meta-synthesis was conducted using the Joanna Briggs Institute meta-aggregation approach. PubMed, CINAHL, Embase, PsycINFO, Scopus, Web of Science, the Cochrane Library, CNKI, Wanfang, VIP, and the China Biomedical Literature Database were searched from inception to April 25, 2026. Two reviewers independently screened studies, extracted data, and assessed methodological quality using the JBI Critical Appraisal Checklist for Qualitative Research. Synthesized findings were assessed using the JBI ConQual approach.

**Results:**

Six qualitative studies involving 113 registered nurses were included. Thirty-eight findings were extracted and aggregated into eight categories, which generated three synthesized findings: (1) GAI may enhance work efficiency and professional competence; (2) nurses encounter ethical, cultural, and operational challenges when using GAI; and (3) nurses require training, institutional support, and clear guidance for standardized GAI use while maintaining positive expectations for future applications.

**Conclusion:**

The available qualitative evidence suggests that registered nurses perceive GAI as a potentially supportive tool for improving efficiency, assisting clinical and research decision-making, and promoting professional development. However, the current evidence base remains limited, and the findings should be interpreted as preliminary. Further research across diverse healthcare systems and cultural contexts is needed to clarify how GAI can be safely and responsibly integrated into nursing practice.

**Systematic review registration:**

https://www.crd.york.ac.uk/PROSPERO/view/CRD420261365306, Identifier CRD420261365306.

## 1 Introduction

Generative artificial intelligence (GAI) is increasingly being integrated into healthcare (1). Large language models (LLMs) such as ChatGPT have shown potential in clinical decision support, nursing documentation, and research assistance (2, 3). In China, the National Health Commission has issued policy guidance to promote the deep application of AI in nursing management, clinical decision-making, and patient services (4). Registered nurses, as the primary workforce in clinical settings, are central to determining whether GAI can be safely and effectively integrated into nursing practice (5). However, the use of GAI also raises concerns regarding content accuracy, misinformation, data privacy, and academic ethics (6, 7). Although qualitative studies have explored nurses’ experiences with GAI in various contexts, most have been confined to single settings or specific populations, lacking a systematic synthesis (8, 9). Moreover, differences in healthcare systems and cultural contexts limit the direct applicability of findings from one country to another (10). In this review, artificial intelligence (AI) is used as an umbrella term referring to computational systems that perform tasks requiring human-like intelligence. Generative artificial intelligence (GAI) is a subset of AI that can generate new content, such as text, images, audio, or other outputs, based on user prompts and training data. Large language models (LLMs) are a major type of text-generating GAI designed to process and generate human language. ChatGPT is a specific LLM-based application and represents one of the most widely discussed GAI tools in healthcare and nursing contexts. Because the included studies mainly focused on text-generating GAI or LLM-based tools, particularly ChatGPT, the findings of this review should be interpreted as primarily applying to text-based GAI systems rather than to all forms of AI. We acknowledge that AI, GAI, LLMs, and ChatGPT differ in technological scope, accessibility, functions, implementation contexts, and user experiences. The following questions guided this review: (1) What are registered nurses’ core experiences of using GAI in clinical and research settings? (2) What challenges do they encounter? (3) What support needs do they express for the safe and effective use of GAI?

## 2 Materials and methods

This qualitative evidence synthesis was reported according to the PRISMA 2020 statement and the ENTREQ guideline for enhancing transparency in reporting qualitative evidence syntheses (11, 12). The review was registered in PROSPERO at the protocol stage (CRD420261365306). No major deviations from the registered protocol occurred in terms of the review question, eligibility criteria, databases searched, or synthesis approach. During revision, the JBI ConQual approach was added to assess the confidence of the synthesized findings and to improve the transparency and interpretability of the evidence synthesis.

### 2.1 Literature search strategy

A systematic literature search was conducted in PubMed, CINAHL, Embase, PsycINFO, Scopus, Web of Science, the Cochrane Library, CNKI, Wanfang, VIP, and the China Biomedical Literature Database from database inception to April 25, 2026. Searches were completed between April 16 and April 25, 2026, and database-specific search dates are provided in Supplementary Appendix A1.

Search terms were developed around three concepts: nurses, generative artificial intelligence, and qualitative research. Because no standardized MeSH term for “generative artificial intelligence” was available at the time of the search, GAI-related concepts were searched using title/abstract terms, including “generative artificial intelligence,” “generative AI,” “GenAI,” “ChatGPT,” “large language model,” “LLM,” and “LLMs.” The reference lists of included studies were also manually screened to identify additional eligible studies.

Full reproducible search strategies for all databases are provided in Supplementary Appendix A1. Gray literature was not systematically searched because this review focused on peer-reviewed qualitative studies with sufficient methodological detail and extractable qualitative findings. This decision is acknowledged as a limitation. Studies published in Chinese or English were included because these were the languages in which the review team could ensure accurate interpretation of qualitative data.

### 2.2 Inclusion and exclusion criteria for literature

Inclusion Criteria: ① Population (P): Practicing nurses (including clinical nurses, registered nurses, and nursing researchers); ② Interest of Phenomenon (I): Nurses’ experiences, perceptions, attitudes, views, and challenges and needs encountered in practice regarding the use of generative artificial intelligence (GAI); ③ Context (Co): Healthcare institutions or nursing research settings; ④ Study Design (S): Qualitative studies (such as phenomenological studies, grounded theory, descriptive qualitative research, narrative research, etc.) or the qualitative component of mixed-methods studies that can be independently separated.

Exclusion Criteria: ① Mixed-methods studies from which qualitative data on nurses’ experiences using GAI cannot be extracted; ② Full text unavailable; ③ Literature not in Chinese or English; ④ Duplicate publications.

### 2.3 Literature screening and data extraction

All retrieved records were exported to EndNote for duplicate removal. Two reviewers independently screened titles and abstracts against the eligibility criteria, followed by full-text review of potentially relevant studies. Disagreements were resolved through discussion or, if consensus could not be reached, by consultation with a third reviewer.

Data were extracted using a standardized Excel form, capturing: first author, publication year, study setting and population, sample size, research method, phenomenon of interest, and main findings.

### 2.4 Evaluation of literature quality

Two researchers, respectively, used the qualitative research authenticity evaluation tool from the Joanna Briggs Institute (JBI) Evidence-Based Healthcare Center in Australia (13) to assess the methodological quality of the included literature. If there were disagreements that could not be resolved through discussion, a third researcher would make the judgment. This evaluation tool contains a total of 10 items, each of which can be rated as “Yes,” “No,” “Unclear,” or “Not Applicable.” Literature that fully meets the evaluation criteria is rated as Grade A, partially meets as Grade B, and does not meet at all as Grade C. This study only included literature with a quality grade of A or B.

### 2.5 Meta-integration method

The results of the studies were integrated using the aggregative integration method in the JBI Evidence-Based Healthcare Center’s meta-integration (14). The researchers repeatedly read the included studies to become familiar with the original findings, participant quotations, author interpretations, and contextual information. Findings were extracted when they directly reflected registered nurses’ experiences, perceptions, challenges, or support needs related to GAI use. Two reviewers independently compared the meanings of the extracted findings and grouped findings with similar meanings into preliminary categories. Disagreements regarding the allocation of findings were resolved through discussion, and a third reviewer was consulted when consensus could not be reached. The preliminary categories were then compared and refined according to conceptual similarity, contextual meaning, and relevance to the review questions. Finally, related categories were aggregated into synthesized findings that represented broader patterns across the included studies. This process generated 38 findings, eight categories, and three synthesized findings.

The confidence of each synthesized finding was assessed using the JBI ConQual approach, which considers dependability and credibility. Dependability was assessed based on the methodological quality of the included studies, particularly the congruity among research methodology, data collection, data analysis, interpretation, and reflexivity. Credibility was assessed according to the extent to which the extracted findings were supported by participant quotations or author interpretations in the primary studies. The ConQual summary of synthesized findings is presented in Table 1.

**Table 1:** ConQual summary of synthesized findings.

Synthesized finding	Contributing evidence	Dependability	Credibility	ConQual score	Rationale for ConQual rating
GAI may enhance work efficiency and professional competence	15 findings; 3 categories; 6 studies	Moderate	Moderate	Moderate	This synthesized finding was supported by evidence across all six included studies and across three categories: improving work efficiency, supporting clinical and research decisions, and promoting professional competence development. Credibility was rated as moderate because the finding was supported by both participant quotations and author interpretations across clinical and research contexts. Dependability was downgraded to moderate because several contributing studies provided limited reporting of researcher reflexivity and cultural or theoretical positioning.
Ethical, cultural, and operational challenges in using GAI	14 findings; 3 categories; 6 studies	Moderate	Moderate	Moderate	This synthesized finding was supported by evidence from all six included studies and across three categories: information accuracy and AI hallucinations, ethical and privacy risks, and cultural and organizational barriers. The finding was relatively consistent across different nursing contexts, including clinical practice, nursing research, neonatal intensive care, oncology nursing, and culturally specific research settings. Credibility was rated as moderate because the evidence was supported by participant quotations and author interpretations. Dependability was downgraded to moderate because several primary studies had methodological limitations, especially limited reflexive reporting and insufficient description of researchers’ cultural or theoretical positioning.
Support needs and positive expectations for standardized GAI use	9 findings; 2 categories; 6 studies	Moderate	Low	Low	This synthesized finding was supported by all six included studies but was based on fewer findings and categories than the first two synthesized findings. The evidence mainly reflected training needs, institutional support needs, expectations for guideline development, and future expectations for GAI applications. Credibility was downgraded to low because these findings were more context-dependent and, in some studies, more future-oriented or expectation-based rather than grounded in extensive direct experience. Expectations for future GAI use may be influenced by organizational resources, cultural context, policy environment, and technological accessibility. Dependability was rated as moderate because the contributing studies had some methodological limitations, particularly limited reporting of reflexivity and cultural positioning.

## 3 Results

### 3.1 Literature search results

**Figure 1:**
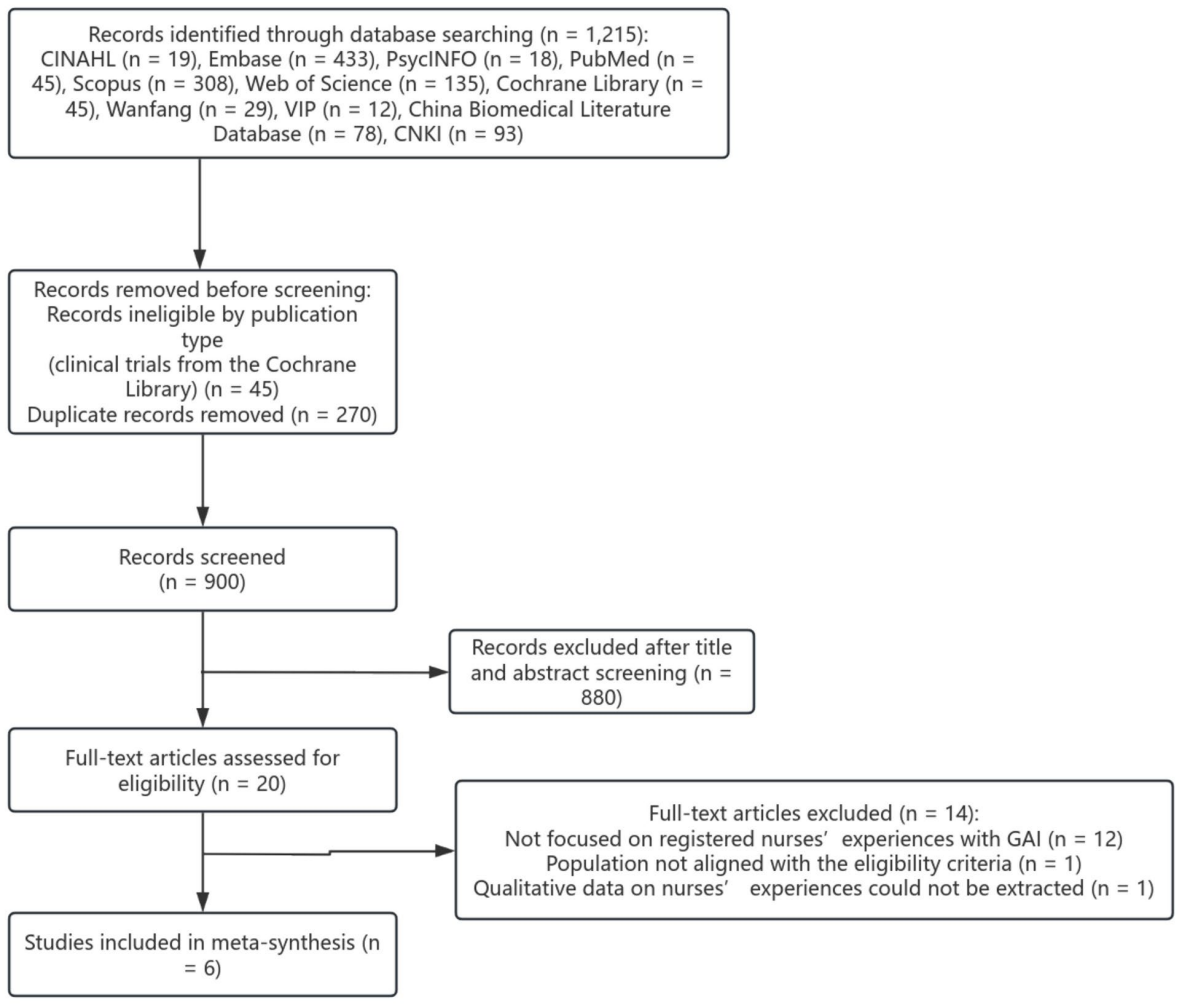
PRISMA flow diagram of the study selection process.

A total of 1,215 records were identified through database searching. Before title and abstract screening, 45 records from the Cochrane Library were removed because they were clinical trials and therefore ineligible by publication type, and 270 duplicate records were removed. The remaining 900 records were screened by title and abstract, of which 880 were excluded. Twenty full-text articles were assessed for eligibility. Fourteen full-text articles were excluded for the following reasons: not focused on registered nurses’ experiences with GAI (*n* = 12), population not aligned with the eligibility criteria (*n* = 1), and qualitative data on nurses’ experiences could not be extracted (*n* = 1). Finally, six studies were included in the meta-synthesis (15–20). The literature screening process is shown in Figure 1.

### 3.2 Basic characteristics of the included literature

The basic characteristics of the included studies are shown in Table 2.

**Table 2:** Characteristics of included studies (*n* = 6).

Included study	Year	Study setting and population	Sample size	Research method	Phenomenon of interest	Main findings
Wang et al. (15)	2025	Maternal and child nursing staff (obstetric nurses, midwives, pediatric nurses) from a tertiary hospital in Beijing, China	16	Descriptive qualitative study; semi-structured interviews	Maternity nursing staff’s experiences of using GAI	3 themes: (1) positive perceived effects of using GAI; (2) constraints in using GAI; (3) needs and expectations for GAI use
Qu et al. (16)	2026	Nursing researchers from three tertiary hospitals in Tianjin, China	14	Descriptive qualitative study; semi-structured interviews	Nursing researchers’ cognition, practical experiences, and ethical considerations regarding the use of GAI in research	4 themes: (1) GAI enhances research efficiency and capability; (2) risks and ethical dilemmas in GAI application; (3) GAI-driven reconstruction of nursing researchers’ capabilities and roles; (4) GAI use standards and professional development pathways
Included study	Year	Study setting and population	Sample size	Research method	Phenomenon of interest	Main findings
Tong et al. (17)	2025	Clinical nurses from 11 provinces, 2 autonomous regions, and 3 municipalities in China, all of whom had participated in a three-day “GAI for Nursing Research” training program	23	Descriptive qualitative study; focus group interviews	Clinical nurses’ perceptions and experiences of GAI-assisted research training	4 themes: (1) utilization efficacy; (2) research facilitation; (3) role reversal; (4) positive expectations
Alruwaili et al. (18)	2025	Neonatal nurses from four high-risk NICUs in the Eastern Province of Saudi Arabia	33	Phenomenological study; semi-structured interviews and focus groups	NICU nurses’ authentic experiences of using GAI in clinical decision-making in high-risk environments	5 themes: (1) clinical decision-making process; (2) professional practice transformation; (3) organizational and system factors; (4) cultural and contextual influences; (5) implementation challenges and solutions
Han et al. (19)	2026	Oncology nurses (from outpatient, inpatient, examination, infection control, and nursing management departments) at the National Cancer Center, South Korea	17	Phenomenological study; semi-structured interviews	Oncology nurses’ cognition, emotions, and readiness for change management regarding GAI application	3 core domains: (1) applicability; (2) implementation considerations; (3) dissemination strategies
Tuppal et al. (20)	2025	Filipino nurse scientists	10	Narrative-focused group discussion; semi-structured interviews	Filipino nurse scientists’ perspectives and future outlook on the application of ChatGPT in research	3 themes: (1) cultural awareness; (2) ChatGPT-specific needs; (3) skills and resource transformation (extended analysis of research nurses)

### 3.3 Methodological quality assessment results of included studies

The methodological quality of six included studies was assessed, among which 2 studies (18, 20) (33.3%) were rated A, and 4 studies (15–17, 19) (66.7%) were rated B, with no C-level studies. Core evaluation items performed well, with main deduction items concentrated on whether ‘the philosophical foundation and methodology are consistent,’ ‘the researcher’s cultural/theoretical stance is clarified,’ and ‘the researcher’s reflection on their own impact is explicit.’ Some studies reported insufficiently on researcher reflexivity and cultural stance. Detailed item-level results of the methodological quality appraisal are provided in Supplementary Table S1. The limited reporting of researcher reflexivity and cultural or theoretical positioning should be understood not only as a methodological reporting limitation, but also as a potential source of interpretive uncertainty. Because nurses’ experiences with GAI involve professional identity, responsibility boundaries, organizational hierarchy, ethical judgment, and trust in emerging technologies, researchers’ own assumptions about nursing practice, technology adoption, and healthcare culture may influence how interview data are collected, interpreted, and represented. Therefore, insufficient reflexive reporting in several primary studies may have affected the credibility and dependability of the original findings and, consequently, the interpretation of the synthesized findings in this review.

### 3.4 Meta-integration results

This study extracted a total of 38 research findings, categorized similar results into 8 new categories, and ultimately integrated them into 3 core findings.

#### 3.4.1 Integration result 1: GAI improves work efficiency and professional competence

##### 3.4.1.1 Category 1: improving work efficiency

Within this synthesized finding, work efficiency and professional competence were treated as related but conceptually distinct categories. “Improving work efficiency” refers mainly to short-term, task-level benefits, such as saving time, reducing repetitive work, accelerating information retrieval, and supporting documentation or research preparation. In contrast, “promoting professional competence development” refers to longer-term changes in nurses’ knowledge, skills, critical thinking, AI literacy, ethical awareness, and capacity for professional growth. Although some findings were relevant to both categories, they were assigned according to their primary meaning and emphasis in the original studies.

Participants in the included studies described that GAI could reduce the time required for repetitive paperwork, information retrieval, and daily tasks (“I need to make a health education board for babies, and it can help me do a hotspot analysis, outline a draft, and then I can supplement it professionally, which improves efficiency” (15); “It helps me answer patients questions more quickly and reduces repetitive tasks” (19)). In research scenarios, GAI can also help nurses quickly establish a research framework and shorten the preparation phase [“I have no research experience, but by continuously asking GAI questions, it can help me set up a research framework in 1 or 2 h. If I tried to do it on my own, it would take several days” (17)].

##### 3.4.1.2 Category 2: supporting clinical and research decisions

Participants described GAI as an auxiliary decision-making tool, providing directional guidance without replacing professional judgment [“The AI points me in a direction, but I still make the call” (18)]. In addition, GAI can quickly organize literature and data to support research work [“AI can help me quickly organize literature and data” (16)], and it demonstrates unique value in integrating localized medical data and public health research [“ChatGPT can be trained on Filipino medical research data … to better suit the specific needs of Filipino nurse scientists” (20)].

##### 3.4.1.3 Category 3: promoting professional competence development

The GAI can drive the enhancement of nurses’ professional strengths and overall literacy [“driving professional strengths and overall literacy improvement” (15)]. At the same time, GAI stimulates innovative thinking and provides interdisciplinary perspectives, helping to break through traditional research approaches [“when you propose an initial idea, it can offer unexpected variables or design ideas, a capability far beyond traditional search tools” (16)]. In addition, the use of GAI also encourages nurses to proactively improve technical expertise, enhance ethical awareness, and expand resource development capabilities [“Nurse scientists would benefit from training in data analysis techniques tailored explicitly to AI tools” (20)].

#### 3.4.2 Integration result 2: ethical, cultural, and operational challenges in the use of GAI

##### 3.4.2.1 Category 4: information accuracy and AI hallucinations

The included studies suggest that participants maintained a cautious attitude toward the accuracy and reliability of GAI-generated content [“I feel that the professional answers it provides cannot be completely relied upon … if there are no clear sources, it should not be used casually” (15)]. GAI may generate fake references [“They look very standard, but are often false, and must be verified one by one, otherwise it could lead to academic accidents” (16)]. Therefore, nurses emphasize that GAI outputs need to be validated in combination with local medical data and professional judgment [“ChatGPT outputs need to be critically evaluated to ensure they do not promote a one-size-fits-all approach” (20)].

##### 3.4.2.2 Category 5: ethical and privacy risks

Participants expressed concerns that the use of GAI may involve privacy breaches [“There are concerns, for example, will it leak my privacy?” (15)]. If medical data is accessed or altered by multiple people, it could harm patient privacy [“If everyone can easily access or modify patient data, it will harm the personal privacy of patients” (18)]. In addition, nurses are confused about the academic ethical boundaries of GAI [“Where is the boundary for using it to modify words and generate entire passages? Should it be actively declared? These questions lack clear guidance” (16)]. Based on this, nurses call for strengthened training and institutional safeguards regarding patient privacy, data security, and ethical standards [“Training in ethical considerations of using AI in health research is essential” (20)].

##### 3.4.2.3 Category 6: cultural and organizational barriers

The included studies suggest that nurses’ experiences of GAI use were influenced by cultural adaptability and organizational support [“Our system values senior physician input. Even if the AI suggests something insightful, I must present it respectfully, acknowledging our hierarchy” (18)]. GAI should be trained in the local language and cultural context [“ChatGPT should be trained on diverse datasets that reflect different cultural backgrounds and languages” (20)]. Nurses report that the prevalence of GAI is limited and that organizational training support is insufficient [“In the nursing profession, I feel the exposure is relatively narrow, and I only find out if recommended by friends or relatives” (15); “Without adequate learning, we cannot identify technical errors” (18)].

#### 3.4.3 Integration result 3: support needs and positive outlook for GAI applications

##### 3.4.3.1 Category 7: training and institutional support needs

Participants reported a need for systematic training on GAI usage and the establishment of clear institutional regulations and policy safeguards [“Training related to GAI needs to be carried out so that we know how to use it, what can be used, and what cannot be used” (15)]. Nursing research personnel suggest that academic institutions and journals should formulate transparent and practical GAI usage guidelines and ethical standards, including data traceability requirements and author responsibility definitions [“Promptly establish transparent and practical GAI usage guidelines and ethical standards, including data traceability requirements and author responsibility definitions” (16)]. Oncology nurses indicate that, in addition to technical training, a complete legal protection and feedback mechanism is needed to ensure clear responsibility and promote safe usage [“If the responsibility falls entirely on the nurses, they may not use it” (19)].

##### 3.4.3.2 Category 8: positive expectations for future development

Despite facing multiple challenges, nurses remain positive about the development prospects of GAI in the nursing field, anticipating the emergence of smarter, safer, and more adaptable tools. Oncology nurses hope that GAI can deeply integrate with electronic medical record systems in the future to achieve more precise personalized care (19). NICU nurses look forward to GAI playing a greater role in home care, health education, and public health scenarios (18). Filipino nurse scientists emphasize that the development of GAI should align with the core values of nursing [“By proactively engaging with the potential of ChatGPT and advocating for its ethical integration … ensure that the technology aligns with the core values of nursing” (20)]. Across the included studies, participants tended to view GAI as an auxiliary tool to enhance professional capabilities rather than a technology to replace clinical judgment.

### 3.5 Confidence in synthesized findings

The confidence levels of the three synthesized findings ranged from low to moderate, as shown in Table 1. For each synthesized finding, the ConQual assessment considered both dependability and credibility. Dependability was downgraded when contributing studies had methodological limitations, particularly limited reporting of researcher reflexivity and cultural or theoretical positioning. Credibility was downgraded when the supporting evidence was derived from fewer findings or categories, when findings were supported mainly by author interpretations rather than rich participant quotations, or when the evidence was closely related to specific organizational, cultural, or policy contexts. The third synthesized finding was rated as low because it was supported by fewer findings and categories and reflected more context-dependent expectations regarding future GAI implementation, training, institutional support, and governance.

## 4 Discussion

Across the three synthesized findings, nurses’ experiences with GAI can be interpreted as a process of negotiated adoption rather than simple acceptance or rejection. The synthesis suggests that nurses evaluated GAI not only according to its perceived usefulness in reducing workload, supporting decision-making, and facilitating research, but also according to whether its use could be reconciled with professional accountability, trust in AI-generated knowledge, role boundaries, cultural fit, institutional governance, and the preservation of human-centered care. In this sense, the central insight generated by this meta-synthesis is that GAI implementation in nursing is not merely a technical issue of tool availability or usability. Rather, it is a socio-technical and professional negotiation in which nurses balance potential empowerment against risks to safety, ethics, responsibility, and nursing values (21–23).

### 4.1 The bidirectional value of GAI in enhancing work efficiency and professional competence

The integrated results suggest that participants in the included studies perceived GAI as having potential bidirectional value in clinical and research work: it may reduce repetitive paperwork and information retrieval time, provide directional support in decision-making processes, and promote professional competence development. This is consistent with the findings of Saban and Dubovi (2) indicating that GAI has potential empowerment effects in nursing practice. GAI effectively alleviates nurses’ time pressure by automatically generating documentation frameworks, integrating information, and offering multi-perspective ideas (24). However, nurses still emphasize the primacy of their own judgment when using GAI [e.g., “AI points me in a direction, but I still make the call” (18)], suggesting that GAI’s value lies in “empowerment” rather than “replacement.” This technological positioning may stem from nurses’ high sense of responsibility for clinical decision safety and the irreplaceability of human-centered care (25, 26). Notably, related surveys show that while up to 97.1% of clinical nurses hold a positive attitude toward GAI, only 7.39% have received formal training (27), indicating a potential capability gap in technology usage. This implies that without systematic training and ethical guidance, the practical application of GAI may face safety and operational risks. Therefore, nursing managers should regard GAI as a collaborative tool, strengthen AI ethics and critical thinking training in continuing education (28), and establish clear human-computer collaboration processes and responsibility delineation (25), to ensure that GAI enhances efficiency without undermining professional judgment and nursing quality. From the perspective of technology acceptance theories, these findings may be interpreted through perceived usefulness (22, 23). Nurses were more likely to view GAI positively when they perceived that it could reduce workload, support decision-making, and improve research efficiency. However, perceived usefulness alone is insufficient for sustained adoption. Facilitating conditions, including training, institutional guidance, and workflow integration, are also necessary for nurses to use GAI safely and confidently (23).

### 4.2 Ethical, cultural, and operational challenges in using GAI require attention

Across the included studies, participants described concerns about information accuracy, AI hallucinations, privacy and security risks, blurred academic ethical boundaries, cultural adaptation, and insufficient organizational support when using GAI. This aligns with the findings of Hu et al. (29) regarding nurses’ perception of multidimensional dilemmas in the broader use of AI. The black-box nature of GAI and the uncontrollability of its training data are the technical roots causing information distortion and “hallucination” phenomena (30), while ethical risks such as data privacy, algorithmic bias, and unclear accountability are particularly prominent in nursing contexts (7). Surveys indicate that nurses are highly sensitive and anxious about issues like GAI-generated “fake references,” potential data leaks, and unclear accountability (31). Moreover, in different cultural contexts (such as the Philippines and Saudi Arabia), traditional values like family involvement and respect for older adults place higher demands on the cultural adaptability of GAI (18). Based on these challenges, it is recommended that relevant institutions accelerate the development of guidelines and ethical standards for the use of GAI in nursing contexts (25), incorporate feedback from multidisciplinary teams and frontline nurses during AI system development, and strengthen nurses’ central role and critical thinking training when using GAI (28), in order to promote coordinated improvement in cultural adaptation, data security, and organizational support. These challenges also reflect perceived risk, which is a key factor influencing technology adoption. Concerns about AI hallucinations, privacy leakage, unclear accountability, and cultural mismatch may reduce nurses’ trust in GAI, even when they recognize its potential usefulness. From a socio-technical perspective, the safe implementation of GAI in nursing depends not only on technological performance but also on ethical governance, organizational culture, professional accountability, and compatibility with nursing workflows (21).

### 4.3 Nurses’ support needs and future expectations for the standardized application of GAI

The integrated results show that nurses have a strong demand for systematic training in GAI, organizational system guarantees, and policy support, while holding positive expectations for its future development, which is consistent with the research of Rony et al. (32). The survey shows that although 97.1% of clinical nurses have a positive attitude toward GAI, only 7.39% have received formal training (27), highlighting the urgency of building a training system. Nurses’ focus has shifted from “whether the technology will replace me” to “how to use it within the rules,” especially regarding the clarity of “usage boundaries” and “responsibility delineation” (16). At the same time, large language models still face multiple ethical challenges in nursing applications, such as data privacy and security, algorithmic bias, educational equity, informed consent, and ambiguous responsibility assignment (33). Although 70% of nursing journals have policies regarding LLMs, differences still exist between journals (34), indicating the necessity of unified standards.

Based on the above-mentioned needs, it is recommended to incorporate GAI ethics, data security, and prompt design into continuing education courses (35), promote a ‘human-machine collaborative’ nursing education and practice model (36, 37), accelerate the formulation of operational standards for clinical and research applications (38), and enhance nurses’ professional abilities through management system support and critical thinking training (28). Across the included studies, participants tended to regard GAI as a tool to enhance professional capabilities rather than a means to replace clinical judgment (39). Compared with previous broad artificial intelligence studies, this study further reveals nurses’ particular concern regarding ‘usage boundaries’ and ‘responsibility delineation’ in the context of generative artificial intelligence. The findings suggest that GAI implementation in nursing should be understood as a socio-technical process (21). Nurses’ willingness and ability to use GAI are shaped by the interaction among individual competence, technological design, organizational support, ethical standards, and cultural context. Therefore, future implementation strategies should not focus solely on introducing GAI tools but should also establish clear training systems, responsibility boundaries, feedback mechanisms, and culturally responsive governance frameworks.

By integrating findings across clinical, research, and culturally diverse nursing contexts, this review provides a more nuanced understanding of GAI adoption in nursing. The findings suggest that nurses’ willingness to use GAI depends on the interaction between perceived functional benefits and professional, ethical, organizational, and cultural conditions. Therefore, successful GAI implementation requires more than technical training. It also requires trustworthy AI outputs, transparent responsibility allocation, culturally responsive design, institutional governance, and mechanisms that protect nurses’ professional judgment and human-centered care. This interpretation extends existing discussions of GAI in healthcare by highlighting nursing-specific concerns related to accountability, relational care, role boundaries, and practice-based trust.

### 4.4 Transferability and contextual limitations

The transferability of these findings should be interpreted cautiously. Only six studies involving 113 registered nurses were included, and four of the six studies were conducted in China or involved predominantly Chinese nurses. This geographical concentration may have shaped the synthesized findings, particularly those related to regulatory concerns, academic use of GAI, organizational support, and expectations for training. In addition, the included studies were conducted in diverse contexts, including maternal and child nursing, neonatal intensive care, oncology nursing, and nursing research. Nurses’ experiences with GAI may differ according to clinical specialty, organizational culture, technological accessibility, and national regulatory environments. Therefore, the findings should be understood as preliminary and context-specific rather than as generalizable conclusions about nurses globally.

Researcher reflexivity is also important for interpreting the transferability of the synthesized findings across cultural contexts. In studies of GAI use, participants’ accounts may be shaped by local expectations about professional authority, institutional hierarchy, patient privacy, academic integrity, and the nurse–patient relationship. If primary researchers do not clearly report their cultural positioning, theoretical assumptions, or potential influence on data interpretation, it becomes more difficult to determine whether the findings mainly reflect participants’ experiences, researchers’ interpretations, or context-specific assumptions about technology and nursing roles. This limitation is particularly relevant in a geographically concentrated evidence base, where cultural norms regarding respect for authority, family involvement, organizational decision-making, and trust in digital technologies may influence how nurses describe both the benefits and risks of GAI. As a result, the synthesized findings should be interpreted with attention to the reflexive and cultural limitations of the primary studies.

## 5 Conclusion and implications

This meta-synthesis provides preliminary qualitative evidence on registered nurses’ experiences of using GAI in clinical practice and nursing research. The available evidence suggests that GAI may support nurses by improving perceived work efficiency, assisting clinical and research decision-making, and promoting professional development. However, the evidence base remains small and context-specific, and the findings should be interpreted cautiously.

Several limitations should be acknowledged. First, only six qualitative studies involving 113 registered nurses were included, which limits the breadth of the evidence base. Second, four of the six included studies were conducted in China or involved predominantly Chinese nurses, which may affect the transferability of the findings to other healthcare systems and cultural contexts. Third, only Chinese and English publications were included, which may have introduced language bias. Fourth, gray literature was not systematically searched, and only peer-reviewed publications were included; therefore, potential publication bias cannot be excluded. Fifth, several included studies provided limited reporting on researcher reflexivity and cultural or theoretical positioning. This may have influenced not only the credibility and dependability of the primary findings, but also the way nurses’ experiences with GAI were interpreted across different cultural and organizational contexts.

Finally, GAI technologies are rapidly evolving, and experiences with different tools, such as ChatGPT and localized large language models, may differ across clinical and research settings. At the practical level, nursing managers should establish routine training and feedback mechanisms for GAI use. Policymakers and healthcare institutions need to improve governance frameworks for data security, ethical review, and responsibility attribution. Nursing educators should consider AI literacy, prompt design, ethical awareness, and critical appraisal of AI-generated outputs as emerging competencies for nurses. Future studies should include larger, multi-regional, and longitudinal qualitative or mixed-methods designs to explore how nurses interact with different GAI systems across diverse healthcare contexts.

## Data Availability

Publicly available datasets were analyzed in this study. This meta-synthesis analyzed data from previously published studies, all of which are cited in the manuscript and are publicly available through their respective journals. No new primary data were generated for this study.
