# Supplementary material for: Registered nurses’ experiences with generative artificial intelligence: a meta-synthesis of qualitative studies
Source: Front Public Health. 2026 Jul 27;14:1917065. doi: 10.3389/fpubh.2026.1917065 (PMC13454418; doi:10.3389/fpubh.2026.1917065)
Supplement: Supplementary file 1 [file Data_Sheet_1.pdf]

Supplementary Appendix A1 | Full search strategies

PubMed

Search date: April 16, 2026

#1: ("Nurses"[Mesh]) OR "Nursing Staff"[Mesh]
#2: "Qualitative Research"[Mesh]
#3: (((((Nurse[Title/Abstract]) OR (Nursing Personnel[Title/Abstract])) OR (nursing staff[Title/Abstract])) OR (Personnel, Nursing[Title/Abstract])) OR (Nurse, Registered[Title/Abstract])) OR (Registered Nurse[Title/Abstract])
#4:)AND ("generative artificial intelligence"[Title/Abstract] OR "Artificial Intelligence, Generative"[Title/Abstract] OR "Gen AI"[Title/Abstract] OR GenAI[Title/Abstract] OR "Generative AI"[Title/Abstract] OR Chatbot[Title/Abstract] OR Chat-GPT[Title/Abstract] OR "Chat GPT"[Title/Abstract] OR ChatGPT[Title/Abstract] OR "large language model"[Title/Abstract] OR "large language models"[Title/Abstract] OR LLM[Title/Abstract] OR LLMs[Title/Abstract] OR Bard[Title/Abstract] OR Gemini[Title/Abstract] OR Claude[Title/Abstract])
#5: ((((((((Qualitative Research[Title/Abstract]) OR (feeling[Title/Abstract])) OR (cognition[Title/Abstract])) OR (attitude[Title/Abstract])) OR (expectation[Title/Abstract])) OR (psychology[Title/Abstract])) OR (belief[Title/Abstract])) OR (viewpoint[Title/Abstract])) OR (qualitative[Title/Abstract])) OR (interviews[Title/Abstract])) OR (grounded theory[Title/Abstract])) OR (phenomenology[Title/Abstract])) OR (mixed methods[Title/Abstract])
#6: #1 OR #3
#7: #4
#8: #2 OR #5
#9: #6 AND #7 AND #8

Embase

Search date: April 20, 2026

#1:('nurse'/exp OR 'nurse':ti,ab OR 'anaesthesist nurse assistant':ti,ab OR 'anesthetist nurse assistant':ti,ab OR 'community health nurse':ti,ab OR 'community health nurses':ti,ab OR 'nurse, community health':ti,ab OR 'nurses':ti,ab OR 'nurses, community health':ti,ab OR 'nurses, public health':ti,ab OR 'nursing assistance':ti,ab OR 'public health nurse':ti,ab OR 'public health nurses':ti,ab)
#2:('artificial intelligence'/exp OR 'generative artificial intelligence':ti,ab OR 'creative AI':ti,ab OR 'creative artificial intelligence':ti,ab OR 'gen-AI':ti,ab OR 'genAI':ti,ab OR 'generative A.I.':ti,ab OR 'generative AI':ti,ab)
#3:('qualitative research'/exp OR 'qualitative research':ti,ab OR 'feeling':ti,ab OR 'cognition':ti,ab OR 'attitude':ti,ab OR 'expectation':ti,ab OR 'psychology':ti,ab OR 'belief':ti,ab OR 'viewpoint':ti,ab OR 'qualitative':ti,ab OR 'interviews':ti,ab OR 'grounded theory':ti,ab OR 'phenomenology':ti,ab OR 'mixed methods':ti,ab)
#4: #1 AND #2 AND #3

Cochrane

Search date: April 21, 2026

#1 (Nurse* OR "Registered Nurse" OR "Registered Nurses" OR "Nurse, Registered" OR "Nurses, Registered" OR "Nursing Personnel" OR "Personnel, Nursing" OR "Nursing Staff"):ti,ab,kw

#2(qualitative OR "qualitative research" OR interview* OR "grounded theory" OR phenomenology OR (mixed NEXT method*) OR feeling* OR cognition* OR attitude* OR expectation* OR psychology* OR belief* OR viewpoint* OR experience* OR perception* OR perspective* OR emotion* OR view* OR understand* OR narrative* OR "lived experience"):ti,ab,kw

#3("generative artificial intelligence" OR "Generative AI" OR GenAI OR "Gen AI" OR ChatGPT OR "Chat GPT" OR "Chat-GPT" OR ChatGPTs OR Chatbot* OR "Artificial Intelligence, Generative" OR (large NEXT language NEXT model*) OR LLM OR LLMs OR "Bard" OR "Gemini" OR "Claude"):ti,ab,kw

#4 #1 AND #2 AND #3

CINAHL

Search date: April 20, 2026

(Nurse* OR "Registered Nurse" OR "Registered Nurses" OR "Nurse, Registered" OR "Nurses, Registered" OR "Nursing Personnel" OR "Personnel, Nursing" OR "Nursing Staff")

AND

("generative artificial intelligence" OR "Generative AI" OR GenAI OR "Gen AI" OR ChatGPT OR "Chat GPT" OR "Chat-GPT" OR ChatGPTs OR Chatbot* OR "Artificial Intelligence, Generative" OR "large language model*" OR LLM OR LLMs OR Gemini)

AND

(qualitative OR "qualitative research" OR interview* OR "grounded theory" OR phenomenology OR "mixed method*" OR feeling* OR cognition* OR attitude* OR expectation* OR psychology* OR belief* OR viewpoint* OR experience* OR perception* OR perspective* OR emotion* OR view* OR understand* OR narrative* OR "lived experience")

PsycINFO

Search date: April 21, 2026

(Nurse* or "Registered Nurse" or "Registered Nurses" or "Nurse, Registered" or "Nurses, Registered" or "Nursing Personnel" or "Personnel, Nursing" or "Nursing Staff").ti,ab,id.

("generative artificial intelligence" or "Generative AI" or GenAI or "Gen AI" or ChatGPT or "Chat GPT" or "Chat-GPT" or ChatGPTs or Chatbot* or "Artificial Intelligence, Generative" or "large language model*" or LLM or LLMs).ti,ab,id.

(qualitative or "qualitative research" or interview* or "grounded theory" or phenomenology or "mixed method*" or feeling* or cognition* or attitude* or expectation* or psychology* or belief* or viewpoint* or experience* or perception* or perspective* or emotion* or view* or understand* or narrative* or "lived experience").ti,ab,id.

Scopus

Search date: April 23, 2026
TITLE-ABS-KEY (nurse* OR "registered nurse" OR "registered nurses" OR "nursing personnel" OR "nursing staff") AND TITLE-ABS-KEY ("generative artificial intelligence" OR "generative AI" OR GenAI OR "Gen AI" OR ChatGPT OR "Chat GPT" OR "Chat-GPT" OR chatbot* OR "large language model*" OR LLM OR LLMs) AND TITLE-ABS-KEY (qualitative OR "qualitative research" OR interview* OR "grounded theory" OR phenomenology OR "mixed method*" OR experience* OR perception* OR perspective* OR attitude* OR cognition* OR feeling* OR emotion* OR belief* OR viewpoint* OR narrative* OR "lived experience")

Web of Science
Search date: April 16, 2026
TS = (nurse* OR "registered nurse" OR "registered nurses" OR "nursing personnel" OR "nursing staff") AND TS = ("generative artificial intelligence" OR "generative AI" OR GenAI OR "Gen AI" OR ChatGPT OR "Chat GPT" OR "Chat-GPT" OR chatbot* OR "large language model*" OR LLM OR LLMs) AND TS = (qualitative OR "qualitative research" OR interview* OR "grounded theory" OR phenomenology OR "mixed method*" OR experience* OR perception* OR perspective* OR attitude* OR cognition* OR feeling* OR emotion* OR belief* OR viewpoint* OR narrative* OR "lived experience")

Chinese databases

CNKI

Search date: April 22, 2026

Search fields: Title/Abstract/Keywords
Search strategy:(护士 OR 护理人员 OR 临床护士 OR 注册护士 OR 护理科研人员) AND(生成式人工智能 OR 生成式 AI OR 人工智能生成内容 OR AIGC OR ChatGPT OR 大语言模型 OR LLM OR LLMs)AND(体验 OR 感知 OR 认知 OR 态度 OR 看法 OR 观点 OR 心理 OR 情绪 OR 需求 OR 挑战 OR 质性研究 OR 质性访谈 OR 访谈 OR 半结构式访谈 OR 焦点小组 OR 现象学 OR 扎根理论)

Wanfang Database

Search date: April 25, 2026

Search fields: Title/Abstract/Keywords
Search strategy:(护士 OR 护理人员 OR 临床护士 OR 注册护士 OR 护理科研人员) AND(生成式人工智能 OR 生成式 AI OR 人工智能生成内容 OR AIGC OR ChatGPT OR 大语言模型 OR LLM OR LLMs)
AND(体验 OR 感知 OR 认知 OR 态度 OR 看法 OR 观点 OR 心理 OR 情绪 OR

需求 OR 挑战 OR 质性研究 OR 质性访谈 OR 访谈 OR 半结构式访谈 OR 焦点小组 OR 现象学 OR 扎根理论)

VIP Database

Search date: April 23, 2026

Search fields: Title/Abstract/Keywords
Search strategy:(护士 OR 护理人员 OR 临床护士 OR 注册护士 OR 护理科研人员) AND(生成式人工智能 OR 生成式 AI OR 人工智能生成内容 OR AIGC OR ChatGPT OR 大语言模型 OR LLM OR LLMs)AND(体验 OR 感知 OR 认知 OR 态度 OR 看法 OR 观点 OR 心理 OR 情绪 OR 需求 OR 挑战 OR 质性研究 OR 质性访谈 OR 访谈 OR 半结构式访谈 OR 焦点小组 OR 现象学 OR 扎根理论)

China Biomedical Literature Database (CBM)

Search date: April 23, 2026

Search fields: Title/Abstract/Keywords
Search strategy:(护士 OR 护理人员 OR 临床护士 OR 注册护士 OR 护理科研人员) AND(生成式人工智能 OR 生成式 AI OR 人工智能生成内容 OR AIGC OR ChatGPT OR 大语言模型 OR LLM OR LLMs)AND(体验 OR 感知 OR 认知 OR 态度 OR 看法 OR 观点 OR 心理 OR 情绪 OR 需求 OR 挑战 OR 质性研究 OR 质性访谈 OR 访谈 OR 半结构式访谈 OR 焦点小组 OR 现象学 OR 扎根理论)
