# Supplementary material for: Registered nurses’ experiences with generative artificial intelligence: a meta-synthesis of qualitative studies
Source: Front Public Health. 2026 Jul 27;14:1917065. doi: 10.3389/fpubh.2026.1917065 (PMC13454418; doi:10.3389/fpubh.2026.1917065)
Supplement: Supplementary file 3 [file Data_Sheet_3.pdf]

Supplementary Table S1 | Methodological quality appraisal of included studies using the JBI Critical Appraisal Checklist for Qualitative Research

Included study	Q1	Q2	Q3	Q4	Q5	Q6	Q7	Q8	Q9	Q10	Overall quality
Wang ZT et al.	Unclear	Yes	Yes	Yes	Yes	Yes	Yes	Yes	Unclear	Yes	B
Qu TX et al.	Unclear	Yes	Yes	Yes	Yes	Yes	No	Yes	No	Yes	B
Tong L et al.	Unclear	Yes	Yes	Yes	Yes	Yes	No	Yes	No	Yes	B
Alruwaili AN et al.	Yes	Yes	Yes	Yes	Yes	Yes	Yes	Yes	Yes	Yes	A
Han J et al.	Yes	Yes	Yes	Yes	Yes	Unclear	Yes	Yes	Unclear	Yes	B
Tuppal CP et al.	Yes	Yes	Yes	Yes	Yes	Yes	Yes	Yes	Yes	Yes	A

Note. Q1: Congruity between the stated philosophical perspective and the research methodology; Q2: Congruity between the research methodology and the research question or objectives; Q3: Congruity between the research methodology and the methods used to collect data; Q4: Congruity between the research methodology and the representation and analysis of data; Q5: Congruity between the research methodology and the interpretation of results; Q6: Statement locating the researcher culturally or theoretically; Q7: Influence of the researcher on the research, and vice versa, addressed; Q8: Adequate representation of participants and their voices; Q9: Ethical approval or ethical considerations reported; Q10: Conclusions drawn from the analysis or interpretation of the data.
